# A primer on sequencing and genotype imputation in cattle

**DOI:** 10.1093/jas/skaf293

**Published:** 2025-08-28

**Authors:** Troy N Rowan

**Affiliations:** Department of Animal Science, University of Tennessee, Knoxville, TN, USA37996; Department of Large Animal Clinical Sciences, University of Tennessee College of Veterinary Medicine, Knoxville, TN, USA37996

**Keywords:** cattle, GWAS, genomic prediction, imputation, sequencing

## Abstract

Since their introduction to the beef industry, genotyping technologies have opened the door for genomic selection and accelerated population improvement. Single-nucleotide polymorphism arrays have served as the backbone of genomic selection programs since their introduction. Developments in sequencing and genotype imputation offer alternatives to array-based genotyping that have the potential to provide orders of magnitude more information at a lower cost. In this review, I give an overview of array-based genotyping, genomic sequencing, low-pass sequencing, and imputation. Additionally, I provide perspectives on how low-coverage sequencing and imputation could simultaneously drive genomic discovery and be used for routine genotyping as sequencing costs continue to decline.

## Teaser

This paper reviews historical and recent developments in genotyping and genomic sequencing technologies in cattle. It also provides considerations for genetic evaluations interested in leveraging the next generation of sequencing-based genotyping approaches.

## Introduction

Widescale access to genotyping is one of the most important innovations integrated into animal breeding programs over the last 50 yrs (García-Ruiz et al., 2016). Routine genotyping has become a stalwart in purebred cattle populations and is rapidly expanding into the commercial sector. Applications of genomic technologies have evolved to encompass a variety of applications, from parentage verification (McClure et al., 2018) to marker-assisted selection tools (Van Eenennaam et al., 2007) to genetic abnormality screens (Charlier et al., 2008) to the backbone of genomic selection programs (Meuwissen et al., 2001). These applications have evolved alongside new technologies and approaches for capturing the genetic makeup of animals.

Genotyping in cattle populations was initially performed with restriction fragment length polymorphisms, followed by variable-length microsatellites, which were integral to early genome-mapping studies (Ihara et al., 2004; Womack, 2012). The creation of a high-quality reference genome sequence (Elsik et al., 2009) enabled the development of high-density single-nucleotide polymorphism (SNP) arrays (Van Tassell et al., 2008; Matukumalli et al., 2009), opening the door to implementing genomically enhanced predictions of genetic merit (Meuwissen et al., 2001; VanRaden, 2008). As genotyping reached the wider industry, decreasing costs was a major priority. To date, this has primarily been accomplished through reductions in marker numbers using the same general genotyping platform (Wu et al., 2016). Recent developments in sequencing technologies and genotype imputation algorithms present attractive alternative approaches to array-based genotyping (Li et al., 2021).

In this paper, I briefly review the evolution of array-based genotyping and sequencing in beef cattle applications. I then cover the concept of genotype imputation for both array-based genotypes and low-coverage genome sequencing data. Finally, I discuss the promise of low-coverage sequencing and imputation in genetic evaluations and provide some considerations for maximizing its utility. While the applications of these technologies are broad and far-reaching, my primary focus will be on applications related to genetic prediction in beef cattle.

## Array-based Genotyping

Array-based genotypes have been the workhorse of genomic-enhanced evaluations since their introduction (Matukumalli et al., 2009). These arrays delivered the molecular platform needed to implement the foundational concepts described in (Meuwissen et al., 2001). Meuwissen, Hayes, and Goddard showed that due to extensive linkage disequilibrium (LD) throughout the bovine genome, a relatively small fraction of carefully chosen markers could account for all of the effective segments that exist within a population (Goddard and Hayes, 2009; Pocrnic et al., 2016). This enabled multiple approaches that modeled marker effects to predict the aggregate genetic merit for individuals across the genome.

These genotyping arrays lean on an underlying knowledge of where and how frequently SNPs exist across the genome. The design of SNP arrays relies on a discovery stage that selects an optimal set of variants from either a known database of variation or a discovery population of sequenced individuals (Van Tassell et al., 2008). Probes are designed for the flanking sequences of these locations so that a one-base extension with a fluorescently labeled nucleotide can be read and interpreted as the nucleotide on that strand at the site of the known SNP (Rampal, 2008).

SNP arrays are designed with variants that fit 4 main criteria. First, to maximize information, arrays tend to choose SNPs that have moderate-to-high minor allele frequencies (MAF). This ensures that maximum variability can be observed with a limited subset of markers, though it may result in difficulty representing low MAF genomic features or causal variants via LD. Other criteria include a generally uniform distribution of markers across the genome, proximity to known causal variants, and locations relative to genes (Fan et al., 2010). One major drawback of SNP arrays is that the discovery process may lead to ascertainment bias in the final SNP set. This results in an array where not all markers are representative or informative in populations that were not a part of the discovery set.

The first widely available commercial SNP array in the United States was the Illumina BovineSNP50 BeadChip, which contained around 50,000 markers. Other array platforms were subsequently developed that increased marker density for association mapping studies (Illumina Bovine HD—777,000 + SNPs) or for assaying a greater proportion of functional areas of the genome (Rowan et al., 2019; Xiang et al., 2021). Other efforts focused on reducing costs strategically reduced marker set sizes (Boichard et al., 2012; Wu et al., 2016) while still capturing sufficient genetic variation for use in genomic predictions (Rolf et al., 2010).

The implementation of widespread genotyping and genomic selection was rapidly adopted in the dairy industry (García-Ruiz et al., 2016) as it immediately replaced sire testing programs aimed at identifying high genetic merit young bulls (Hayes et al., 2009). Adoption was slower in beef populations, but some breeds are now leveraging hundreds of thousands of genotypes in routine genetic evaluations (Retallick et al., 2022). There is also increasing interest in generating genomic predictions for commercial cattle for use in heifer selection decisions and feeder cattle marketing (Akanno et al., 2019; Arisman et al., 2020; DeLong et al., 2023).

Despite their widespread usage, array-based genotypes do have some important drawbacks. Considerable effort and resources are needed for discovery and design. Furthermore, the cost of synthesizing new pools of probes or updating marker sets is time-consuming and expensive. Most importantly, the costs associated with arrays are failing to drop at the same rate as genomic sequencing (Wetterstrand, 2013). With broader applications to commercial cattle on the horizon, necessary declines in cost will be difficult to achieve via arrays (Newton and Berry, 2020).

## Sequencing 101

Modern genotyping and sequencing approaches are built on the knowledge of variations at specific physical locations in the genome. Understanding where variations exist in physical, chromosomal space enables the mapping of quantitative trait loci (QTL) for complex traits and the design of high-throughput genotyping tools (Rhie et al., 2021). Early karyotyping and mapping studies initiated these efforts in cattle and culminated in the completion of the first cattle reference genome (Womack, 2012). This initial draft genome was generated from a single purebred Hereford Female from the Line 1 population housed at the USDA-ARS research station in Fort Keough, Montana (Bovine Genome Sequencing and Analysis Consortium et al., 2009). This cow, Dominette, was identified as being particularly inbred, which made the process of assembling segments of the genome more straightforward by minimizing heterozygosity. Innovations in genome assembly and sequencing platforms have resulted in more highly contiguous and resolved reference genomes (Koren et al., 2018; Rosen et al., 2020). Ongoing efforts aim to integrate genome diversity from multiple breeds, lines, or related species into a comprehensive “pangenome” (Smith et al., 2023).

Sequencing is the chief method used to capture the DNA content of an individual’s genome (Shendure et al., 2017). Multiple technologies exist for reading genomic sequences. These platforms all differ in their accuracy, read length, and price. These approaches and their relative tradeoffs are reviewed at length by (Goodwin et al., 2016). Long-read approaches have gained popularity in recent years, as they can generate moderately accurate reads that span large areas of the genome, making assembly easier and allowing for the detection of large structural rearrangements (Amarasinghe et al., 2020). Despite improvements to long-read technologies, most routine sequencing applications today use short-read platforms (e.g., Illumina) that provide high-accuracy reads at a relatively low cost. DNA sequencing relies on shearing the DNA into smaller chunks, sometimes amplifying with PCR, and then using a sequencing platform to capture 100 to 200-base pair reads at a time (Bentley et al., 2008).

Unless being used to generate a de novo assembly (Li and Durbin, 2024), reads are typically aligned to a relevant reference genome using a sequence alignment tool such as the Burrows-Wheeler Aligner (Li and Durbin, 2009). Once reads are aligned to the reference genome, genotype calling algorithms identify likely variants in an individual’s genome relative to the reference. These variants can generally be grouped into three main classes that include single-nucleotide polymorphisms (SNPs, one-base pair difference between individual and reference), insertions/deletions (INDELs, <50 bp segments inserted or deleted in individual relative to the reference), or larger structural variants (SVs, large-scale rearrangements, copy number variants, etc.).

**Figure 1.**
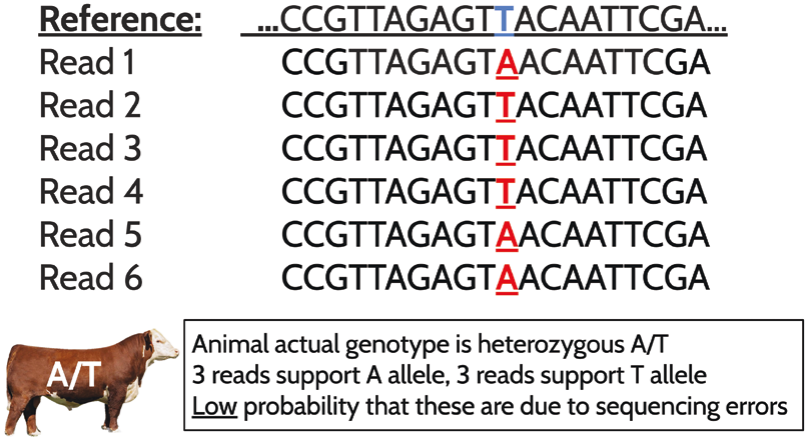
Example scenario representing sequencing coverage at a heterozygous locus. In this example, an A/T SNP is present in an animal. The top string of bases represents the haploid reference genome. Supporting reads 1 to 6 build evidence that the position is heterozygous in the animal of interest.

Sequencing is not perfectly accurate, and errors can occur during the sequencing process. Error rates differ based on the sequencing platform, but for most short-read approaches tend to occur between 0.1% and 0.5% depending on the instrument (Pfeiffer et al., 2018; Stoler and Nekrutenko, 2021). An increased depth of sequencing reads across a base is essential for distinguishing false variant calls and observing heterozygous genotypes (Sims et al., 2014). **Figure 1** shows an example of a SNP at a position receiving additional support as a true heterozygote as subsequent reads accumulate. Each supporting read for an alternative allele is essential, as the likelihood that multiple errors at the same position occur due to sequencing issues is exceedingly low. Additionally, the random sequencing of chromosomes means that reads will not evenly cover both alleles for a heterozygous site. Additional coverage of these sites will result in increased confidence in calling heterozygote genotypes.

We define sequencing coverage as the average number of reads that cover a given base in the genome. Sequencing coverage can be calculated by multiplying the number of reads by the length of the read and dividing that number by the size of the genome. For example, 150 million 200-base pair reads across a bovine genome of approximately 3 billion base pairs would result in an average coverage of 10×. It is essential to remember that coverage metrics are genome-wide averages and are not calculated for individual bases. Sequencing an animal to 10× coverage means that some sites may have less than two reads at some positions and more than 20 in others. Average coverage and filtering based on read depth will largely depend on the tolerance for possible false variant calls in a sequencing project.

## Phasing and Genotype Imputation

Beyond genomic predictions, population-level genotyping has empowered research to link genetic variants to complex traits (Goddard and Hayes, 2009). Early QTL mapping studies that used microsatellites relied on small numbers of samples and markers, resulting in low power and a lack of genomic resolution (Haley, 1995; Khatkar et al., 2004). These challenges severely limited the ability of mapping studies to detect causal variants, particularly when effect sizes were small and traits were highly polygenic (Ron and Weller, 2007). Following the introduction of genotyping platforms, genomic datasets quickly gained sufficient power to perform genome-wide association studies (Risch and Merikangas, 1996; Pearson and Manolio, 2008). Genome-wide association studies use mixed linear models to identify associations between alleles and complex phenotypes (Zhang et al., 2010). The adoption of genotyping led to a rapid expansion of the QTL catalog and played essential roles in identifying informative variants for use in genetic evaluations (Hu et al., 2013).

This expansion of the QTL catalog did not, however, result in the identification of variants that were truly causal. Genotype arrays have sufficient marker content for genomic prediction applications. The evenly spaced markers of the BovineSNP50 are an average of 60 kb apart. Depending on the population sampled, variants may occur at a rate of one every 100 bases (Hoff et al., 2017), though this number will increase with population diversity and sample number as novel variants are identified. This discrepancy means that unless a marker is very close to a causal variant, the linkage between the 2 may not be strong enough to result in a statistically significant association (Spencer et al., 2009). Further, the more common MAF spectra of SNP arrays may result in a failure to detect QTL signal from rare causal variants (Auer and Lettre, 2015).

Sequencing all animals from a phenotyped population to high coverage is not an economically practical solution for mapping studies where statistical power is primarily derived from larger sample sizes (Hayes, 2013; Yengo et al., 2022). We can, however, use sequenced individuals to statistically infer missing genotypes through the process of imputation (Marchini and Howie, 2010). Genotype imputation algorithms use statistical approaches, typically Hidden Markov Models (HMM), to infer genotype states at missing sites based on a high-resolution set of reference haplotypes (Das et al., 2018). These could be in the form of a denser SNP array, such as the Illumina Bovine HD, or a set of high-coverage sequenced animals, such as the 1,000 Bull Genomes Project (Hayes and Daetwyler, 2019).

**Figure 2.**
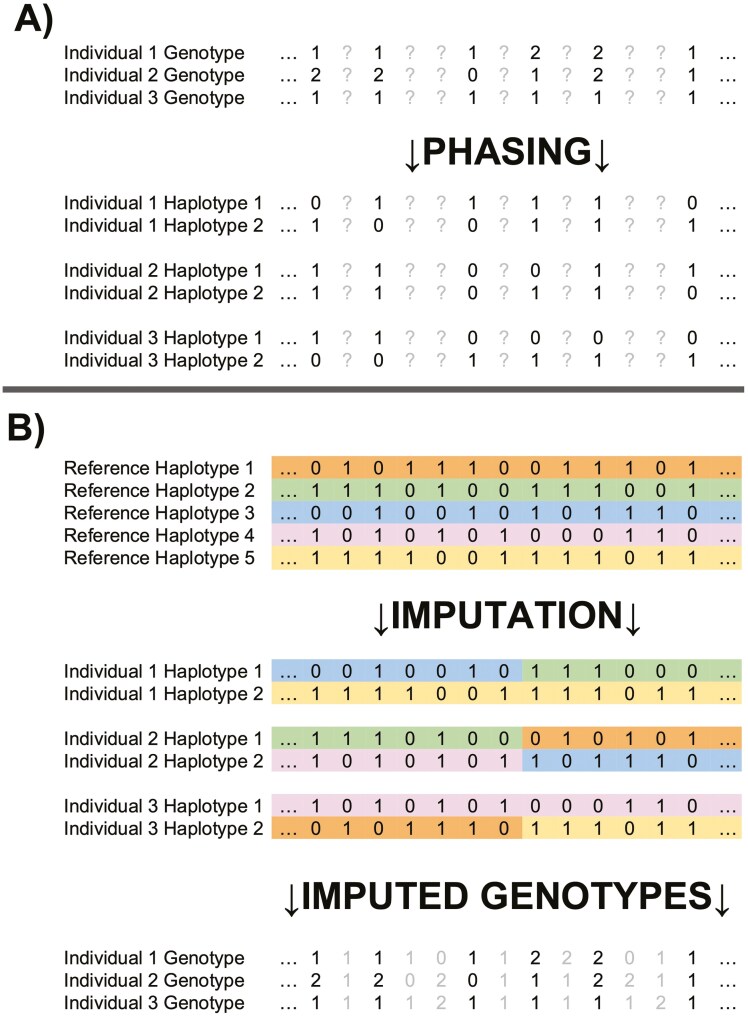
Overview of chip-based imputation. (A) Shows the process of computational phasing as additive genotypes (alternate allele counts) are phased onto contiguous haplotypes. (B) Shows the inference of missing variants from haplotypes onto phased genotypes, followed by the complete set of imputed genotypes.

Imputation leverages LD (Farnir et al., 2000) to infer missing variants with high accuracy when there is adequate representation of haplotype diversity in a population (Figure 2). While imputation can be performed on unphased data, it is most accurate when genotypes in both the reference (high-density) and target (low-density) datasets are phased and arranged into haplotypes (Daetwyler et al., 2011). Haplotypes are the specific combinations of alleles arranged on a single chromosome (Villa-Angulo et al., 2009). Array genotypes and variant calls from sequencing are, by default, unphased, representing an individual’s additive genotype: 0, 1, or 2 counts of the alternate allele. Computational phasing approaches generally use a combination of the Positional Burrows-Wheeler Transform (PBWT; Durbin, 2014) and HMM (Browning and Browning, 2011) to infer phase from these additive genotypes. These algorithms leverage local correlations of marker state (LD) to identify initial haplotype sets and then refine them iteratively, as described by Li and Stephens (Li and Stephens, 2003). Various phasing approaches, such as BEAGLE (Browning et al., 2021), Eagle (Loh et al., 2016), and SHAPEIT (Hofmeister et al., 2023), implement these algorithms slightly differently to provide additional increases in phasing accuracy and computational speed-ups.

Inferred haplotypes can then be used in downstream imputation to infer missing target genotypes present in the reference. The most frequently used HMM-based imputation algorithms for array data include BEAGLE (Browning et al., 2018), IMPUTE (Rubinacci et al., 2020), and MACH (Li et al., 2010; Das et al., 2016). Livestock datasets differ from human datasets because they usually include extensive pedigree data for genotyped animals. This familial information is leveraged by imputation algorithms such as FImpute (Sargolzaei et al., 2014) and findhap (VanRaden et al., 2013).

Genotype imputation, like sequencing, is an imperfect process where errors can be introduced (Li et al., 2009). Imputation errors occur when an imputed marker does not match an individual’s true genotype. When used in association analyses, imputation errors directly lead to reduced statistical power as they break real associations between causal variants and markers. This break in associations typically results in false negatives rather than false positives. (Lau et al., 2024). There are multiple strategies for empirically measuring imputation accuracy in studies where both actual and imputed genotypes are known. The most straightforward is genotype concordance, which is the proportion of correctly imputed genotypes for either an individual or a single variant across a group of individuals. Concordance tends to inflate the imputation accuracy of rare variants, as an imputation algorithm that simply guesses the major allele in the population is likely to correctly impute by chance (Ramnarine et al., 2015). A related statistic, the Imputation Quality Score (IQS), adjusts the concordance rate for low-frequency variants, providing a measure of genotype correctness that is not inflated for rare SNPs (Lin et al., 2010). The squared correlation (r^2^) between imputed genotype dosages and the genotypes called in testing individuals similarly deflates accuracies at rare alleles by effectively penalizing incorrect guesses at rare alleles (Das et al., 2018).

Empirical accuracy calculations are essential for understanding the quality of imputation approaches in an experimental setting, but are not practical for routine imputation scenarios where actual sequence-level genotypes are not known. Imputation software generally takes two approaches to estimating accuracy. First, they may implement these same empirical calculations for masked variants that are genotyped in the target population (comparing actual vs. imputed genotypes). Second, they can use posterior genotype probabilities to estimate the squared correlation for markers not typed on the input data. These approaches are reviewed extensively by Das et al. (2018 and (Das et al., 2016). Estimates of imputation accuracy for non-typed markers are helpful in performing filtering prior to downstream association analyses. The r^2^ value can be interpreted as a reduction in statistical power, where 1-r^2^ is effectively the reduction in power to detect a true association at an imputed marker. The r^2^ filter used in a study may vary, but is generally above 0.3 (Das et al., 2018).

Multiple factors can be responsible for reducing the accuracy of imputation. Imputation is built on the assumption that a limited number of haplotypes exist in a population. For accurate imputation to occur, the underlying haplotypes from a population must be represented in the reference panel used. The most common cause for imputation errors is a lack of representation in the reference panel. This occurrence is particularly a challenge for rare variants as they are inherently less likely to be present in a subset of animals (Fernandes Júnior et al., 2021). Even with large reference panels, imputation accuracies (r^2^) for rare variants (MAF < 0.01) can drop to 0.80 or lower, even when common variants are imputed with extremely high accuracies (r^2^ > 0.99; Rowan et al., 2019). This is not a major issue for imputing between array densities, as they are, by default, biased towards more common variants. Problems arise when imputing sequence variants, as variation across the genome is overwhelmingly rare (Daetwyler et al., 2014). Some evidence exists that leveraging pedigree information may help improve rare variant imputation accuracy, though this idea has not been explored deeply in livestock populations (Ullah et al., 2018).

Larger-scale representation issues can occur when a breed or line with which the target individual shares ancestry is omitted from the reference panel (Rowan et al., 2019). The pattern matching for successful imputation requires that a haplotype be observed in the reference panel in order to be correctly imputed into the target. As such, increased diversity in reference panels generally results in higher imputation accuracies (Bouwman and Veerkamp, 2014; Brøndum et al., 2014). Rowan et al. (2019) showed that a multi-breed reference panel improved imputation accuracies, particularly for admixed populations, populations with open herdbooks, and populations with large amounts of shared ancestry to other breeds. The majority of these accuracy gains occurred for low MAF variants. In the same study, breeds with adequate numbers of high-density animals within the breed did not experience any decrease in imputation accuracy when other breeds were introduced to the reference panel. Some other studies with smaller reference panels and more homogenous target populations have observed slight decreases in imputation accuracy (Lloret-Villas et al., 2023; Kamprasert et al., 2024).

In addition to reference panel diversity, imputation accuracy can be impacted by a variety of other causes, including phasing accuracy, LD patterns, and variant call quality in the reference. Phasing errors occur when false recombination events are inferred by a phasing algorithm that do not exist on an individual’s actual chromosomes (Choi et al., 2018). These phasing errors complicate imputation algorithms that perpetuate these errors onto subsequent steps. Work by Li and colleagues (2023) showed that the correctness of genotype calls and allele phase in the reference panel is as important as reference panel composition in delivering accurate imputation (Li et al., 2023).

Starting assay density also affects overall imputation quality, with higher-density assays experiencing more accurate imputation (Deng et al., 2021). When imputing genotypes from commercially available arrays with less than 100,000 variants to sequence density, significant improvements in accuracy occur when an intermediate imputation occurs to a higher-density set of SNPs (Korkuć et al., 2019). As such, designing sparser genotyping arrays will further reduce their utility in downstream applications that take advantage of imputation.

## Low-Coverage Sequencing and Imputation

Continued innovations in throughput (Modi et al., 2021) and the introduction of new short-read platforms (Eisenstein, 2023) have resulted in further declines in the price of sequencing (Hu et al., 2021). High-coverage sequencing is attainable for many research studies, but remains too expensive for population-scale genotyping. Genotyping approaches that leverage low-coverage sequencing to perform genotyping are gaining traction in both human and livestock applications.

Sequencing has been applied to genotyping in multiple ways since the widespread availability of Illumina platforms. Early applications focused on using sequencing to capture variation in reduced subsets of the genome. These approaches include the widespread use of genotyping-by-sequencing (GBS) in plants, where specific restriction enzymes are used to greatly reduce the genomic content that needs to be sequenced (Poland and Rife, 2012). This allows sequencing-based approaches to function as an array, where a small but representative proportion of the genome is captured, which is sufficient for use in genomic prediction applications (Poland et al., 2012). Other restriction-based approaches, such as RADSeq, have found utility in delivering cost-effective genotyping to non-model organism species for population genetics studies (Davey and Blaxter, 2010). These approaches were explored in cattle and other livestock populations, though never fully adopted (De Donato et al., 2013; Gorjanc et al., 2015). One advantage of GBS approaches is that they are able to detect population-specific variants that are likely to be missing on SNP arrays (Elshire et al., 2011; Ibeagha-Awemu et al., 2016). GBS approaches require a fraction of the reads needed for high-coverage resequencing of individuals and perform similarly to array-based genotypes in genomic prediction applications (Gorjanc et al., 2015). Still, these GBS approaches fall short in their utility in association mapping and in capturing genotypes at parentage markers or known diagnostic loci.

Work by Pasaniuc and colleagues showed that extremely low-coverage whole-genome sequencing (lcWGS; <0.5×) could be used to accurately infer an individual’s genotypes (Pasaniuc et al., 2012). Even at the time of publication in 2012, their work demonstrated that sequencing-based approaches could generate adequate genotypes at a fraction of the cost of arrays. Due to the nonrandom distribution of sequencing reads, even at very low coverages (<0.5×), some sites will be covered with enough reads to sufficiently distinguish heterozygous genotypes from sequencing errors. Low-coverage sequencing captures the full breadth of genomic diversity, particularly from the low end of the minor allele frequency spectrum, all while avoiding the ascertainment bias associated with array-based genotypes (Lou et al., 2021). Some applications use genotype calls from low-coverage sequencing directly in downstream mapping and genomic prediction applications (Lou et al., 2021), but most pair low-coverage sequencing with an imputation step to more confidently call variants in places that lack sequencing depth. Imputation software designed for lcWGS relies on genotype probabilities from sequencing data rather than hard genotype calls from arrays (Rubinacci et al., 2021). Even at coverages well below 1×, adequate concentrations of reads occur to make high-confidence heterozygote calls for imputation. These probabilities are then used alongside imputation reference panels in HMM-based software like GLIMPSE (Rubinacci et al., 2021, 2023), BEAGLE (Hui et al., 2020), or QUILT (Davies et al., 2021).

The sequence reference panels developed for the imputation of array genotypes are directly portable to lcWGS applications (Snelling et al., 2020). As with arrays, lcWGS imputation relies on having sufficient breed representation in the haplotype reference panel (Lloret-Villas et al., 2023). These approaches have been successfully implemented in cattle populations and have shown similar imputation accuracies to those achieved by starting with array-based genotypes (Zhang et al., 2023). In dairy populations, the accuracies of lcWGS imputation tend to have r^2^ > 0.95 at the sequence-level regardless of imputation software (Teng et al., 2022). Similar challenges exist when imputing rare variation. When applied to variance component estimation (Russell et al., 2023) and breeding value estimation (Snelling et al., 2020), no significant differences existed between the results from lcWGS and array genotypes.

Increased adoption of lcWGS in routine genotyping applications will be driven largely by reductions in cost as new and improved sequencing platforms continue to evolve (Satam et al., 2023). Depth of coverage is the major determinant of imputation accuracy across lcWGS applications. This increase in accuracy comes at the cost of additional sequencing reads. While human applications tend to target ~1× coverage, most studies in cattle and other livestock can achieve high accuracy with coverage between 0.1 and 0.5× (Snelling et al., 2020). The other major cost determinant of lcWGS is library preparation, which is fixed, regardless of sequencing depth. Innovations in miniaturized library preparations and optimized sequencing coverage (~0.1×) could reduce costs further. Taken together, these factors could deliver a sub-$10 genotyping solution in the near future (Li et al., 2021).

One hurdle to implementing lcWGS-imputed genotypes into genetic evaluations is the need to capture markers used for parentage assignment (McClure et al., 2018) and identifying carriers of genetic abnormalities (Whitlock et al., 2008). Approaches exist that allow for the high-coverage (>30×) capture of a subset of genomic loci (Hoff et al., 2022). This ensures maximum accuracy when calling these important markers, as they are highly congruent with array-based genotypes in genetic evaluations.

## Considerations for Genetic Evaluations

Contemporary genetic evaluations in beef breeds include anywhere from tens of thousands to over a million array genotypes (Retallick et al., 2022). Shifting from arrays to lcWGS-imputed genotypes poses a number of logistical and scientific challenges. Chief among these is dataset size. Whereas an array genotype may contain information for 50,000 SNPs per animal, lcWGS will include well over 20 million, a 400-fold increase in information. Initial implementations will likely extract array markers from imputed lcWGS genotypes to allow for equivalent genomic enhancements. In these cases, where a core set of array-based SNPs are being extracted from imputed lcWGS data, no additional information is being contributed to genetic evaluations.

For genetic evaluations that utilize single-step genomic best linear unbiased prediction (ssGBLUP), the marker set used to resolve genomic relationships between individuals is unlikely to experience significant differences from using the total amount of data produced by lcWGS (Jang et al., 2023).

The cost of lcWGS and imputation makes it an immediately appealing discovery tool, capable of generating powerful datasets for association mapping with resolutions that are orders of magnitude higher than from arrays. Using a shared imputation reference panel would even allow studies to combine chip and lcWGS data into a common set of variants. With this combination of resolution and statistical power, GWAS could identify variants in complete LD with causal variants, even those with small effect sizes that are rare within populations (Yengo et al., 2022). For other areas of genomic research, such as studying copy number variation (Kucharík et al., 2021), identifying structural variants (Liu et al., 2023), and performing rare variant association mapping (Martin et al., 2021), lcWGS offers opportunities for discovery while high-coverage sequencing remains prohibitively expensive.

To leverage the variants identified by mapping studies, genetic evaluations would have to adopt strategies that differentially weight functional or putatively causal. When identified and appropriately modeled, these high-information markers have been shown to increase the accuracy of estimated breeding values (MacLeod et al., 2016; Xiang et al., 2019, 2021).

While the imputation of array genotypes is not required for genomic prediction applications, it is a critical step for implementing lcWGS genotyping platforms. Genetic evaluations interested in shifting their genotyping platforms to lcWGS will need to evaluate the state of publicly available sequence reference panels critically. Without sufficient breed representation in these panels, the resulting imputation quality may have negative impacts on genomic predictions. Strategic investments that sequence novel haplotypes will be essential before widespread implementation (Gonen et al., 2017). It will be necessary to focus sequencing efforts on animals that offer the greatest addition of novel haplotypes rather than focusing on highly used sires.

## Conclusions

Advances in genomic technologies have revolutionized genetic evaluation across livestock industries. SNP arrays have served as invaluable tools, but sequencing-based approaches to genotyping are becoming more cost-effective while offering orders of magnitude more information. While not required for using array-based genotypes, imputation is a critical component for making low-coverage sequencing a practical approach for genotyping. Maximizing imputation accuracy requires reference panels that adequately represent the full diversity of haplotypes present in a population. Ongoing construction of these resources will be essential as the industry adopts sequencing approaches. Beyond the potential for cheaper genotyping, genetic evaluations will have to consider how best to leverage the additional information that lcWGS provides as they work to deliver more accurate genomic predictions.

## Abbreviations

SNP: Single nucleotide polymorphism
INDEL: Insertion/deletion
SV: Structural variant
GWAS: Genome-wide association study
LD: Linkage disequilibrium
RFLP: Restriction fragment length polymorphisms
GBS: Genotyping-by-sequencing
lcWGS: low-coverage whole-genome sequencing
ssGBLUP: Single-step genomic best linear unbiased prediction
